# Endothelial Barrier and Its Abnormalities in Cardiovascular Disease

**DOI:** 10.3389/fphys.2015.00365

**Published:** 2015-12-09

**Authors:** Dimitry A. Chistiakov, Alexander N. Orekhov, Yuri V. Bobryshev

**Affiliations:** ^1^Division of Laboratory Medicine, Department of Molecular Genetic Diagnostics and Cell Biology, Research Center for Children's Health, Institute of PediatricsMoscow, Russia; ^2^Laboratory of Angiopathology, Institute of General Pathology and Pathophysiology, Russian Academy of SciencesMoscow, Russia; ^3^Department of Biophysics, Biological Faculty, Moscow State UniversityMoscow, Russia; ^4^Institute for Atherosclerosis Research, Skolkovo Innovation CenterMoscow, Russia; ^5^Faculty of Medicine, School of Medical Sciences, University of New South WalesSydney, NSW, Australia; ^6^School of Medicine, University of Western SydneyCampbelltown, NSW, Australia

**Keywords:** endothelium, endothelial barrier, cell-to-cell junctions, endothelial intercellular junctions, cardiovascular disease

## Abstract

Endothelial cells (ECs) form a unique barrier between the vascular lumen and the vascular wall. In addition, the endothelium is highly metabolically active. In cardiovascular disease such as atherosclerosis and hypertension, normal endothelial function could be severely disturbed leading to endothelial dysfunction that then could progress to complete and irreversible loss of EC functionality and contribute to entire vascular dysfunction. Proatherogenic stimuli such as diabetes, dyslipidemia, and oxidative stress could initiate endothelial dysfunction and in turn vascular dysfunction and lead to the development of atherosclerotic arterial disease, a background for multiple cardiovascular disorders including coronary artery disease, acute coronary syndrome, stroke, and thrombosis. Intercellular junctions between ECs mediate the barrier function. Proinflammatory stimuli destabilize the junctions causing the disruption of the endothelial barrier and increased junctional permeability. This facilitates transendothelial migration of immune cells to the arterial intima and induction of vascular inflammation. Proatherogenic stimuli attack endothelial microtubule function that is regulated by acetylation of tubulin, an essential microtubular constituent. Chemical modification of tubulin caused by cardiometabolic risk factors and oxidative stress leads to reorganization of endothelial microtubules. These changes destabilize vascular integrity and increase permeability, which finally results in increasing cardiovascular risk.

## Introduction

**Figure 1:**
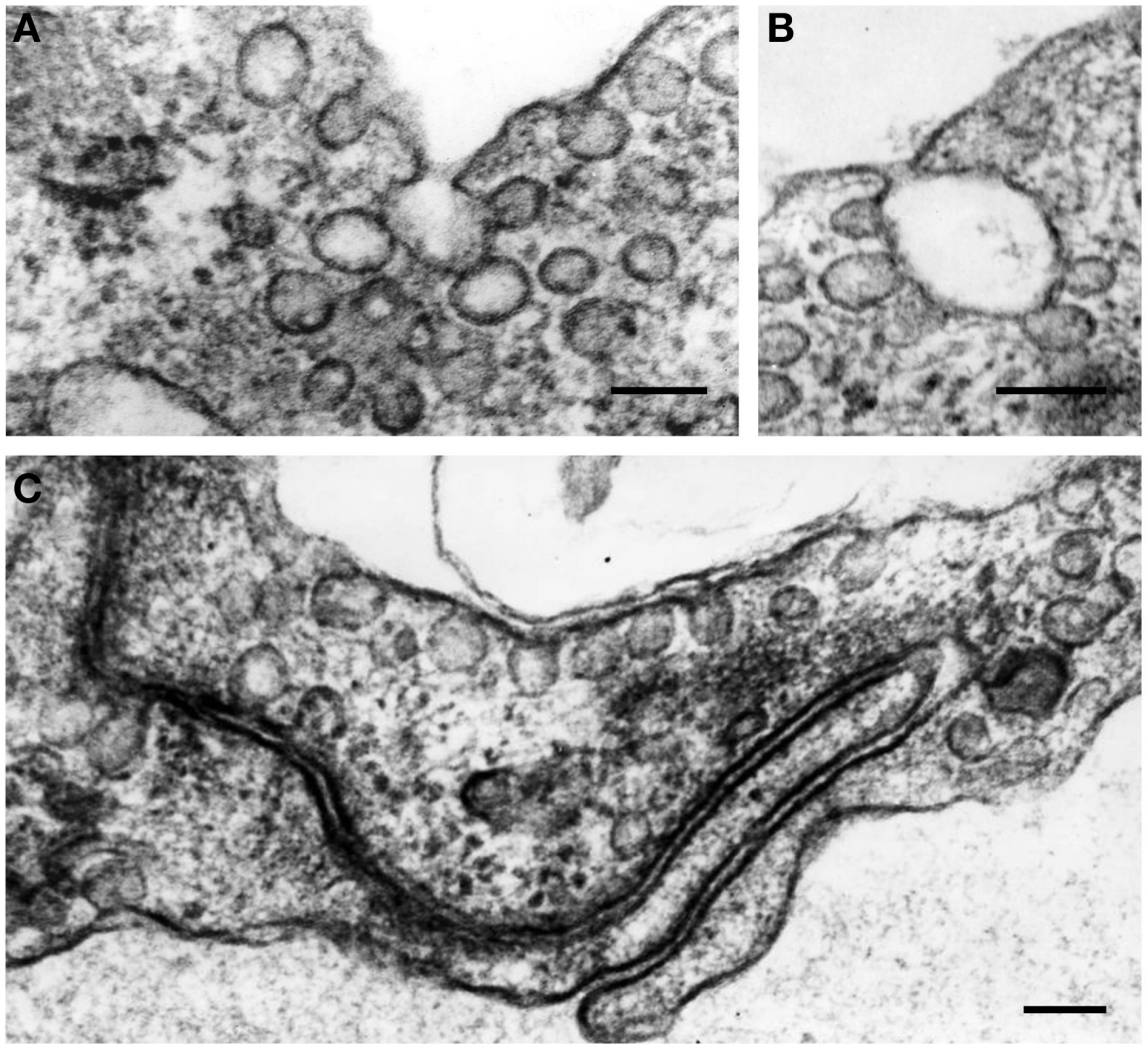
**The formation of vesicles (50–90 nm in diameter) and caveolae along the luminal surface of endothelial cells (A–C)**. In the cytoplasm, vesicles often aggregate and fuse, forming vesicular structures of larger sizes **(A,B)**. Some plasmalemmal vesicles can fuse with cell membrane in the area of EC intercellular contacts **(C)**. Transmission electron microscopy (TEM). Scale bars = 100 nm **(A–C)**. Images are adapted from Bobryshev (1983).

Generally, the endothelium could be defined as a cellular monolayer separating all tissues from the bloodflow (Minami and Aird, 2005; Curry and Adamson, 2010; Chistiakov et al., 2015). In 1950's–60's, implementation of electron microscopy provided rich data about the utlrathin structure of the endothelium. Endothelial cells (ECs) were shown to have some specific cytological characteristics such as presence of Weibel-Palade bodies (Weibel and Palade, 1964). ECs are typically characterized by the presence of large amounts of vesicles and caveolae along the luminal surface, which are capable to move from the luminal to basal surface of ECs, thus providing transendothelial transport of biologically active substances (Figure 1). However, ECs are not uniformly organized. For ECs, a significant phenotypic heterogeneity was shown depending on the location and a vessel type (Repin et al., 1984; Pries and Kuebler, 2006; Dyer and Patterson, 2010; Tse and Stan, 2010). In the vascular system, vascular beds display unique morphological characteristics and functionality, and this contributes to phenotypic endothelial variability (Aird, 2007; Sukriti et al., 2014).

**Figure 2:**
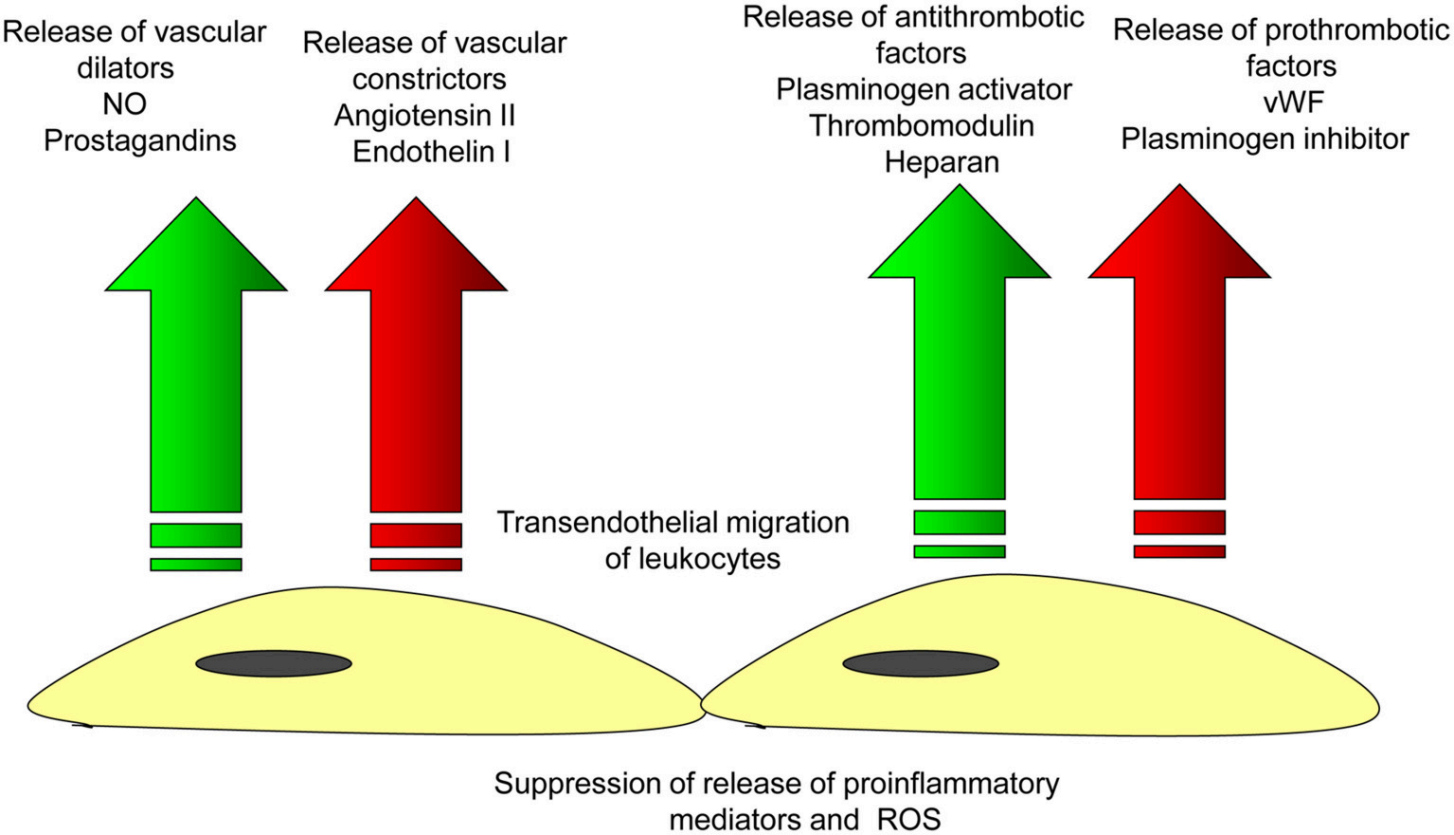
**Endothelial function in the norm**. Arterial endothelial cells are involved in the maintenance of vascular homeostasis by providing balanced release of vasodilatating/vascoconscticting factors and prothrombotic/antithrombotic substances that inhibits the endothelial adhesion of leukocytes and thus, prevents the initiation of vascular inflammation.

The endothelium is responsible for different functions including control of vascular tone and permeability, regulation of vascular inflammation, prevention of thrombosis, and maintenance of vessel integrity (Aird, 2007; Chavez et al., 2011; Figure 2). The vascular integrity and permeability barrier function is crucially supported by intercellular junctions between ECs (Vestweber, 2012). There are two major subtypes of intercellular junctions such as tight junctions (TJ, or zona occludens) and adherens junctions (AJ, or zona adherens; Bazzoni and Dejana, 2004; Hirase and Node, 2012) that could be seen in ECs. Typically, TJ are localized at the apical area of the intercellular cleft. TJ are responsible for barrier function involved in the control of the permeability of solutes between adjacent neighboring cells (Runkle and Mu, 2013). Another TJ property is regulating the lateral protein diffusion within the plasma membrane (van Meer et al., 1986). In venules, endothelial TJ are present irregularly and randomly due to the lack of intensive bloodflow. In ECs of arterioles, TJ are better organized but are tighter than in arteries. In large arteries, ECs exhibit well-structured TJ system because they are exposed to highly pulsatile bloodflow (Aird, 2007).

**Figure 3:**
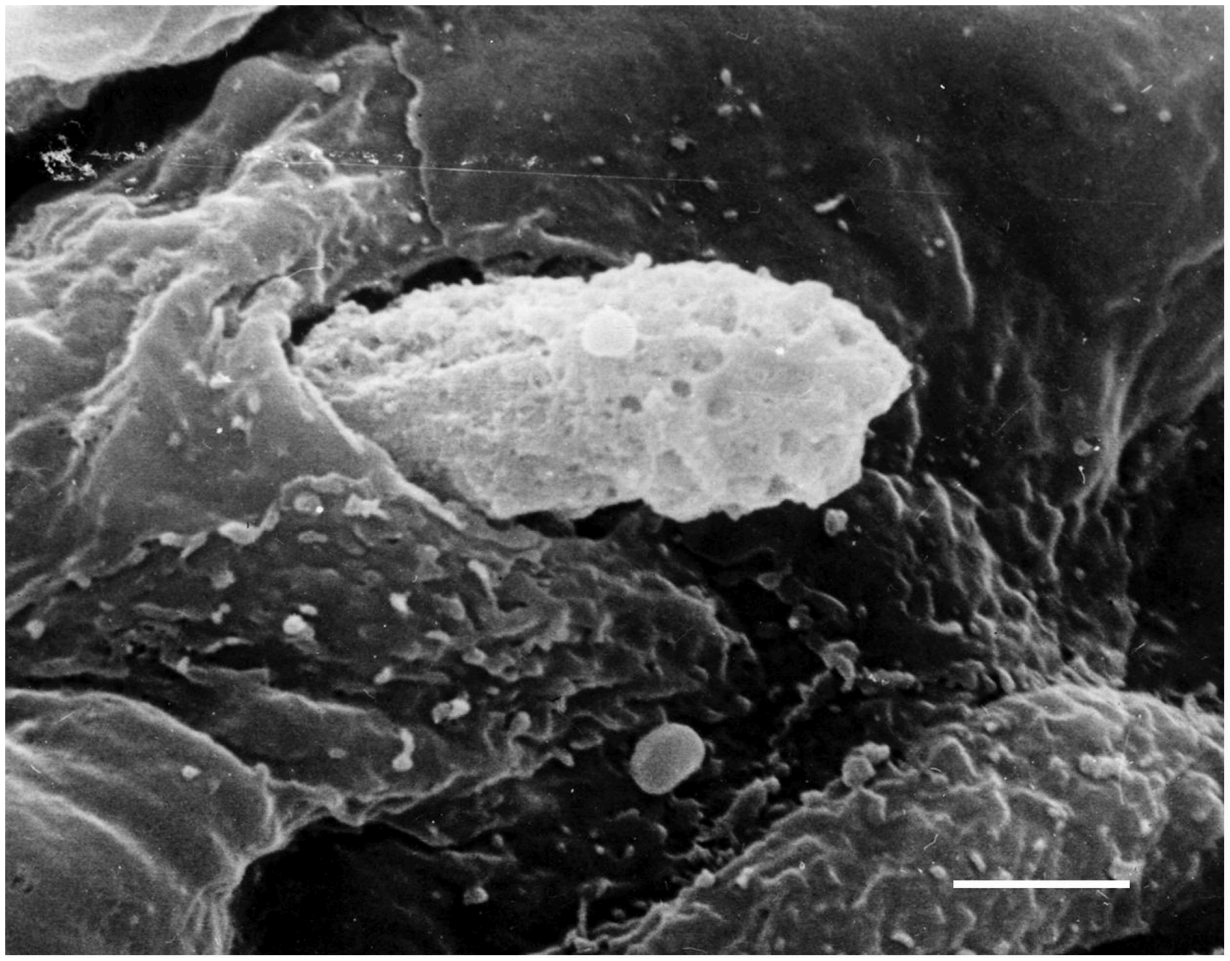
**Penetration of a blood cell through the endothelium into the arterial intima**. Scanning electron microscopy (SEM). Scale bar = 5 μm. Image is adapted from Bobryshev (1983).

In physiological conditions, the barrier function of arterial endothelium is properly regulated and vascular permeability is limited. In vascular pathology such as atherosclerosis, proinflammatory signals activate ECs inducing expression of adhesion molecules and destabilizing the endothelial barrier. This attracts leukocytes including T lymphocytes and monocytes/macrophages and enhances their attachment to the endothelium. Then, leukocytes penetrate the endothelial layer (Figure 3) and infiltrate the arterial intima. In the intima, lymphocytes, and monocytes/macrophages initiate proatherogenic inflammation.

Indeed, in atherosclerotic vessels, the barrier function of ECs is weakened while vascular permeability is significantly increased due to pathological structural changes in intercellular junctions between ECs and loss of the proper regulation of the barrier function. In this review, we will consider structural organization and functional properties of endothelial intercellular junctions in the norm and their alterations in cardiovascular pathology.

## Main protein structural components of endothelial intercellular junctions

**Figure 4:**
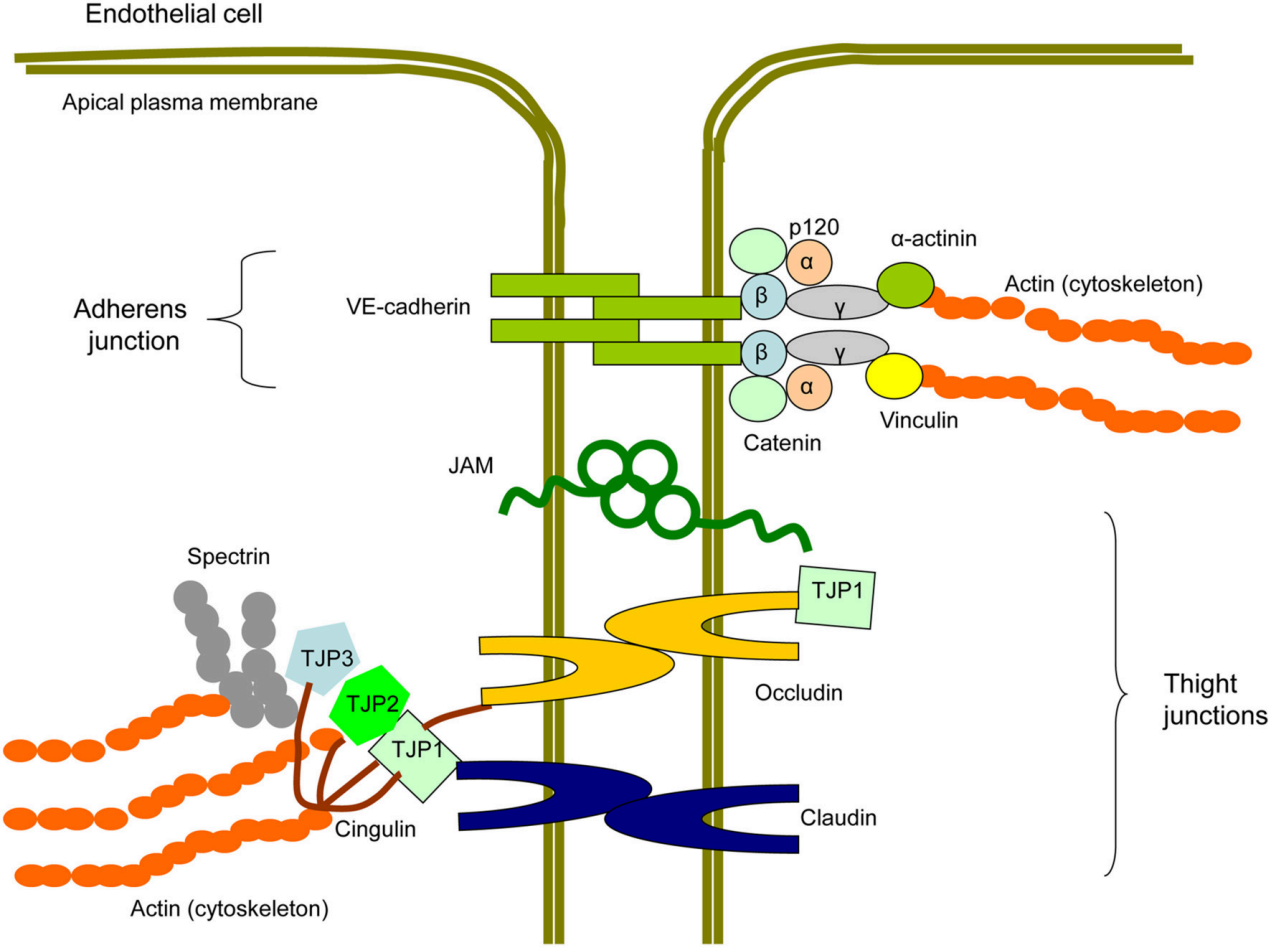
**Scheme of a protein structure of endothelial intercellular junctions (EIJs)**. EIJs consist of tight junctions (TJ) and adherens junctions (AJ) and join two adjacent endothelial cells (Hirase and Node, 2012; Dejana and Orsenigo, 2013). In TJ, membrane proteins are represented by occludin, claudins, and junctional adhesion molecules (JAM). Tight junction proteins (TJP1, TJP2, and TJP3) are cytosolic TJ proteins involved in linking TJ membrane proteins to the cytoskeletal actin. Cingulin is another cytoplasmic TJ protein, which is able to interact with TJPs, occludin, and actin and therefore to link membrane TJ proteins with the cytoskeleton. In AJ, a membrane protein component is represented by vascular-endothelial (VE)-cadherin. p120, β-catenin, and plakoglobin/γ-catenin bind to the C-terminal domain of VE-cadherin. β-catenin interacts with α-catenin that with help of plakoglobin/γ-catenin contributes to the signal transduction from VE-cadherin to the cytoskeleton. α-actinins and vinculain are microfilamentous components that mediate VE-cadherin-dependent mechanotransduction to the cytoskeleton.

Between ECs, intercellular junctions are formed by multiprotein complexes containing transmembrane proteins and cytosol proteins that connect membrane proteins to the intracellular cytoskeleton (Figure 4; Hirase and Node, 2012; Dejana and Orsenigo, 2013). In TJ, membrane-associated proteins are represented by claudins, occludins, and junction adhesion molecules (JAMs; Ebnet, 2008).

Claudins are low molecular proteins (20–27 kDa) that are essential for TJ formation. Claudin is a tetraspan protein that is connected with a cytoskeleton through tight junction proteins (TJP1-4) of which TJP1, TJP2, and TJP3 are the most important for conducting signal transduction in intercellular junctions (Furuse et al., 1998; Morita et al., 1999a,b). Human ECs express claudin-1, −3, −5, −12, and −15 (Morita et al., 1999a,b; Kiuchi-Saishin et al., 2002; Witt et al., 2003; Bélanger et al., 2007). In cultivated human ECs, knockdown of claudin-1 leads to increased TJ permeability (Asaka et al., 2011). In brain ECs, which form the blood-brain barrier, claudin-5 is involved in size-selective barrier function (Nitta et al., 2003). Indeed, claudin-1 and claudin-5 play a central role in the regulation of endothelial TJ permeability.

Human occludin has a 65-kDa mass, and represents a tetraspan integral TJ protein that is a major component of the junctional complex together with claudins. Rather than being important in TJ assembly, occludin is crucial in TJ stability and barrier function (Cummins, 2012). TJP1 is mainly involved in mediating interaction between the COOH-terminus of occludin and cytoskeletal actin (Li et al., 2005). The NH_2_-terminus domain of occludin is involved in TJ sealing and barrier functions. The extracellular loops contribute to the control of paracellular permeability, with the second extracellular loop, which is responsible for location of occludin at the TJ (Feldman et al., 2005). TJs of ECs also contain JAMs that have a single transmembrane domain and two Ig-like domains located in the extracellular part (Martìn-Padura et al., 1998; Hirase and Node, 2012).

Compared to TJ comprising three membrane-associated proteins, AJ have the only a single membrane protein, VE-cadherin (Vascular Endothelial, VE). VE-cadherin has a single transmembrane domain and interacts with another molecule of VE-cadherin expressed on the cell surface of adjacent EC. VE-cadherin is critically involved in the generation of intercellular EC contacts, which are required for angiogenesis and maintaining vessel integrity and barrier function (Dejana et al., 2009; Giannotta et al., 2013; Gavard, 2014).

## Endothelial barrier is involved in the regulation of vessel leakage

Vascular ECs enveloping the vascular lumen represent a border between the blood and extravascular tissues. However, the endothelial barrier is permeable for various molecules and even cells. Ions and soluble solutes could move across ECs in a paracellular manner *via* gaps and transcellular mechanisms (Mehta et al., 2014). Leukocytes transmigrate across the endothelial layer most likely between the cells (Tsukita et al., 2001; Vestweber, 2012; Vestweber et al., 2014). In physiological and pathological conditions, transendothelial trafficking of leukocytes is needed to support immune response, angiogenesis, vascular remodeling, and tissue repair.

Vascular permeability is dynamically regulated in order to keep the tissue homeostasis. Depending on the needs of the organism, the permeability can be increased or reduced through the various regulatory mechanisms and stimuli that influence the strength of EC junctional contacts. Interactions between the structural components of endothelial junctions play a crucial role for supporting a proper barrier function. In endothelial junctions, the membrane density of integral junctional proteins is regulated by vesicular transport proteins *via* internalization and recycling mechanisms (Hirase and Node, 2012).

ADP-ribosylation factor 6 (ARF6) belongs to the family of small GTPases that are key regulators of vesicular transport in eukaryotic cells (D'souza-Schorey and Chavrier, 2006). ARF6 is activated by Pleckstrin and Sec7 domain-containing (PSD), a guanine nucleotide exchange factor (Sakagami, 2008). Activated ARF6 in turn contributes to AJ assembly through the control of E-cadherin internalization in early endosomes (Padovani et al., 2014).

The Rab-small GTPases are involved in the control of TJ-dependent permeability and vesicular transfer of integral junctional proteins. Rab3b and Rab13 are colocolized in TJs and involved to TJ assembly (Weber et al., 1994). Rab13 contributes to mediating the permanent endocytosis of occludin to the plasmalemma of cells (Morimoto et al., 2005). Additionally, Rab13 is implicated in the transfer of claudin-1-containing vesicles from the cytoplasm to intercellular junctions and regulates TJ assembly through protein kinase A (PKA)-mediated signaling mechanism (Köhler et al., 2004; Hirase and Node, 2012). In ECs, Rab5a is responsible for control of claudin-1 localization, a key characteristic of TJ permeability (Asaka et al., 2011). Therefore, Rab GTPase-dependent transfer and location of junctional proteins are involved in regulating the barrier functionality.

Exposure of the vascular endothelium to stressful conditions such as hypoxia and oxidative stress could influence the endothelial permeability. Ischemia and oxidative stress are increased in atherosclerotic vessels and promote endothelial dysfunction through multiple mechanisms including impaired barrier function. In ECs exposed to hypoxia-reoxygenation, relocation of the VE-cadherin-catenin complex led to weakened barrier function. Increase in endothelial permeability was suppressed by endothelial nitric oxide (NO) synthase (eNOS) overproduction in cultured ECs (Ozaki et al., 2002). In EC cultures, treatment with hydrogen peroxide stimulated loss of occludin and cadherin in intercellular junctions suggesting for destabilizing role of reactive oxygen species (ROS)-mediated signaling and oxidative stress on vascular integrity (Kevil et al., 2000).

Extracellular proteases could also regulate vascular permeability. By cleavage of VE-cadherin, thrombin, a blood coagulation pathway inducer, could disturb the endothelial integrity (Rabiet et al., 1996). In VE-cadherin, thrombin cleaves ectodomain followed by further proteolysis with the involvement of γ-secretase and a disintegrin and metalloprotease (ADAM)-10 (Schulz et al., 2008). This mechanism facilitates T cell transmigration through the endothelium (Schulz et al., 2008). ADAM15 mediates transendothelial migration of neutrophils and monocytes *via* the activation of Src/ERK1/2 signaling. However, this metalloproteinase does not digest VE-cadherin or induces its degradation (Sun et al., 2010). In Apolipoprotein E (ApoE)-deficient mice, genetic ablation of ADAM15 resulted in reduction of plaque area by 52% and lesion macrophage infiltration by 69% (Sun et al., 2012). In proinflammatory conditions, activation and accumulation of matrix metalloproteinases in endothelial cell-cell contacts was detected suggesting for their likely involvement to the ablation of the barrier and facilitating leukocyte migration to arterial intima. This pathway could be implicated in the endothelial dysfunction and atherogenesis (Sun et al., 2012).

In human and mouse atherosclerotic lesions, expression of JAM-A is increased (Babinska et al., 2007). In ECs, up-regulation of JAM-A is induced by proinflammatory cytokines (Azari et al., 2011). Up-regulation of JAMs increases adhesion properties of ECs. The extracellular portion of JAMs have a membrane distal VH- and a membrane proximal C_2_-type Ig-like domain capable to bind immune cells (Bradfield et al., 2007). In ApoE-deficient mice, JAM-A inhibition led to reduced neointimal lesion formation and decreased infiltration of the arterial intima media by monocytes (Zernecke et al., 2006). Similarly, in ApoE-deficient mice, inactivation of JAM-C with antibody resulted in significant reduction of neointimal hyperplasia and leukocyte recruitment (Shagdarsuren et al., 2007). In contrast, up-regulation of JAM expression in EC attracted immune cells and facilitated invasion of arterial intima by leukocytes (Garrido-Urbani et al., 2014) thereby indicating the proatherogenic role of JAMs.

## Phosphorylation/dephosphorylation and ubiqiutination as mechanisms of regulation of vascular integrity

Posttranslational modification of junctional proteins is important for proper functioning of the endothelial barrier. Phosphorylation/dephosporylation regulates adhesiveness of VE-cadherin and its interaction with catenins and other cytosolic proteins that link VE-cadherin with actin-based cytoskeleton. A variety of signaling molecules such receptor tyrosine kinases, Src family of tyrosine kinases, and protein tyrosine phosphatases could be observed in AJs (McLachlan and Yap, 2011). E-cadherin was shown to be important for the activation of C-terminal Src kinase (c-Src)-dependent signaling in cell-cell contacts (McLachlan and Yap, 2007; McLachlan et al., 2007). Receptor type-protein tyrosine phosphatase-α (RPTPα) activity is needed to activate E-cadherin-dependent tyrosine-protein kinase CSK (Src) signaling at junctions (McLachlan and Yap, 2011). c-Src activates Src, which in turn recruits phosphatidylinositol-3-kinase (PI3K) to E-cadherin contacts and induces PI3K-dependent signaling cascade (Pang et al., 2005b). PI3K-mediated signaling is one of the major intracellular pathways that regulates many cell functions including cell growth, survival, and intracellular trafficking. On the other hand, RPTPα/Src/Rap1 mechanism is involved in the reciprocal stimulation of E-cadherin-dependent function in junctions by recruiting myosin IIB, a cytoskeletal protein, to the zonula adherens and supporting contractile tension and junctional integrity (Gomez et al., 2015).

Vascular endothelial protein tyrosine phosphatase (VE-PTP) enhances AJ assembly *via* dephosphorylation of VE-cadherin (Nawroth et al., 2002). Down-regulation of VE-PTP or its dissociation from VE-cadherin increases vascular permeability. Binding lymphocytes and neutrophils induces VE-PTP dissociation from VE-cadherin and promotes transendothelial migration (Broermann et al., 2011). Besides leukocytes, vascular endothelial growth factor (VEGF) also stimulates VE-PTP dissociation and increases vascular leakage (Nottebaum et al., 2008).

Phosphorylation/dephosphorylation of junctional proteins is involved in the control of TJ permeability (Staddon, 2001). Lysophosphatidic acid released by activated platelets or histamine induces serine/threonine phosphorylation of occludin resulting in increase of TJ permeability dependent or independent from RhoA/Rho kinase, respectively (Hirase et al., 2001). In hypercholesterolemia, elevated levels of low density lipoprotein (LDL) could weaken the endothelial barrier function by activating Rho while statins decrease permeability by suppressing Rho (van Nieuw Amerongen et al., 2000). These data suggest that RhoA/Rho kinase is a crucial mediator of the endothelial barrier function.

Ras-related C3 botulinum toxin substrate 1 (Rac1) and cell division control protein 42 homolog (Cdc42), both are members of the Rho family of small GTPases, are also implicated in the control of endothelial permeability through regulation of assembly of the actin cytoskeleton (Wojciak-Stothard et al., 2001; Broman et al., 2006). Since Rho is located downstream Cdc42 and Rac1, both Rho-dependent and Rho-independent mechanisms could be implicated in the control of permeability (Hirase and Node, 2012; Huveneers et al., 2015).

Through activating PI3K/Akt pathway, insulin stimulates Rac1, which in turn enhances the barrier function by increasing assembly of the actin-based cytoskeleton through the direct phosphorylation of cortactin, a regulator of interactions between AJ components (Gündüz et al., 2010). Rac1 could stabilize the barrier through the recruitment of the family of p21-activated kinases (PAK), which then activate LIM kinase 1 (LIMK1). PAK cooperate with LIMK1 in LIMK1-mediated inactivation of cofilin, an actin-binding protein that disassembles actin filaments (Dan et al., 2001). Finally, Rac1 can cooperate with Cdc42 in the negative regulation of Ras GTPase-activating-like protein (IQGAP1), which induces dissociation of actin filaments from the catenin-cadherin complex *via* activation of β-catenin (Kuroda et al., 1999). β-catenin in turn prevents association of α-catenin with VE-cadherin junctions (Shapiro and Weis, 2009). Insulin-induced PI3K/Akt-dependent enhancement of the endothelial barrier could also be achieved by Akt-dependent activation of endothelial nitric oxide (NO) synthase (eNOS; Dossumbekova et al., 2008). In diabetes, which is established as a strong independent cardiovascular risk factor, insulin-dependent signaling in ECs is down-regulated due to insulin resistance. Indeed, defects in insulin signaling contribute to diabetes-associated endothelial dysfunction and increased vascular permeability.

The protein ubiqiutination associated with proteasome-mediated protein degradation is crucial for the control of protein modification and turnover. cAMP stimulates expression of Itch, a member of E3 ligase family (Lui and Lee, 2005), which is implicated in occludin ubiqiutination followed by TJ disruption (Traweger et al., 2002). VEGF increases occludin ubiqiutination and promotes TJ fragmentation and interruption of the endothelial barrier (Murakami et al., 2009; Behl and Kotwani, 2015). The ubiquitin-proteasome system (UPS) is involved in the modification and degradation of claudins. The E3 ubiquitin ligase ligand of Numb-protein X1 p80 (LNX1p80) contributes for removal of claudins from TJ (Takahashi et al., 2009). Claudin-5 is modified by UPS-mediated polyubiquitination on lysine 199 followed by proteasome degradation (Mandel et al., 2012). Hypoxia or hypoxia-induced ATP deprivation caused AJ uncoupling and a striking loss of E-cadherin mediated by a proteasome (Bush et al., 2000). Indeed, angiogenic signaling and hypoxia could regulate the vascular permeability with the involvement of UPS.

## Effects of mechanotransduction on endothelial junctions and cytoskeleton

Hemodynamic forces influence the endothelial function especially in arteries where the mechanical stress is significant. Mechanical forces can be transformed to biochemical signals through the mechanisms defined as mechanotransduction (Chatterjee et al., 2015). In blood vessels, endothelial cell-cell contacts and their integrity is the primary target for the mechanotransduction-dependent challenge. In pathology such as muscular dystrophy and cancer, the link between the intercellular junctions and actin cytoskeleton is frequently disrupted or impaired causing the loss of a proper mechanotransduction signal (Jaalouk and Lammerding, 2009).

ECs can sense the type of flow and respond with subsequent changes in cytoskeletal tension and assembly/disassembly of untercellular contacts. For example, laminar flow elevates the tension of the actin cytoskeleton and increases the strength of endothelial cell-cell interactions. The laminar flow (either cyclic strain or steady shear stress) induces Rho GTPase-dependent placement of actin fibers along the flow direction and assembly of junctions (Tzima, 2006). In contrast, perturbed flow disintegrates actin cytoskeleton organization and leads to the AJ disassembly (Ting et al., 2012).

In TJ and AJ, occludin and VE-cadherin respectively were suggested as potential mechanotransducers that sense the bloodflow and contribute to the conversion of mechanical forces to the intracellular signaling (Hahn and Schwartz, 2009). Shear stress effects on EC junctions are not limited by reorganization of the junctional structure and influences on cell-cell contact properties. Hemodynamic forces can regulate expression of junctional proteins. In TJ, low shear stress was shown to down-regulate occludin expression at mRNA and protein level (Conklin et al., 2002, 2007), promote occludin phosphorylation state and decrease vascular integrity (DeMaio et al., 2001). Transient increase in occludin phosphorylation was observed in cultured human umbilical vein endothelial cells (HUVECs) exposed to steady shear stress along with increased hydraulic conductivity (Pang et al., 2005a). By contrast, cyclic strain such as a pulsatile bloodflow (i.e., flow that changes over time in a repetitive manner) was found to increase protein expression of both occludin and TJP1 and occludin mRNA expression. The cyclic strain also stimulated association between occludin and TJP1 and promoted localization of both TJ proteins to the cell-cell contacts thereby increasing the endothelial barrier function (Collins et al., 2006; Colgan et al., 2007).

In endothelial AJ, the VE-cadherin-catenin complex plays a central role in mechanotrasnduction. The cytosolic domain of VE-cadherin interacts with β-catenin or γ-catenin that then recruits α-catenin. α-catenin mediates linking VE-cadherin to actin filaments and play a key role in AJ assembly. Without α-catenin, AJ are disrupted due to the inability of actin to anchor to the VE-cadherin-catenin complex (Gulino-Debrac, 2013). Interestingly, Schulte et al. (2011) developed mice with the VE-cadherin-α-catenin fusion protein that replaced normal VE-cadherin. These mice had extremely stable endothelial AJ that were resistant to leakage-inducing effects of VEGF and histamine and prevented transendothelial migration of leukocytes to inflamed sites. This observation hence indicates that the VE-cadherin-α-catenin hybrid protein associates with cytoskeletal actin with a greater strength compared with wild-type VE-cadherin.

α-Catenin contains three vinculin homology (VH) domains (VH1-3) that are capable to interact with various actin-binding proteins (Maiden and Hardin, 2011). For example, the VH1 domain binds β-catenin while VH3 binds F-actin, TJP-1, and Eplin (Gulino-Debrac, 2013). At high tension, myosin II-actin-mediated stretch can alter α-catenin conformation exposing the vinculin-binding site at the VH2 domain. The cryptic vinculin-binding site is hidden at low tension, and high tension-induced binding of vinculin to α-catenin is required to strengthen the cytoskeletal anchorage to AJ (Shewan et al., 2005).

When force generated by actomyosin machinery was abolished by a myosin II inhibitor, vinculin dissociates from α-catenin and relocates to the focal adhesion plaques, which are located at the ventral surface of ECs and connect the endothelium with the extracellular matrix (ECM; Chervin-Pétinot et al., 2012). Indeed, this finding indicates that association/dissociation of vinculin with α-catenin is regulated by mechanical forces.

While vinculin disappears from AJ upon decreased mechanical stress, Eprin, another actin-binding protein, remains to be associated with α-catenin (Chervin-Pétinot et al., 2012). Eprin belongs to the family of LIM domain-containing proteins and contains a single LIM domain that is essential for protein-protein interactions (Zheng and Zhao, 2007). In the Eprin molecule, the LIM domain resides between two actin-binding domains thereby providing an option for cross-linking binding of actin filaments to eprin and their stabilization (Maul et al., 2003). Since Eprin concomitantly interacts with the VE-cadherin-α-catenin complex and F-actin, this protein is involved in endothelial mechanotransduction (Chervin-Pétinot et al., 2012).

Vinculin contains three domains (head, neck, and tail). In inactive state, the vinculin head domain binds to the tail domain (Ziegler et al., 2006). When actomyosin-mediated stretch induces conformational changes in α-catenin and unmasks the high-affinity vinculin-binding site at the VH2 domain of α-catenin, this disrupts the head-tail interaction and promotes vinculin binding to the opened site (Ishiyama et al., 2013). The neck and tail domains of α-catenin-bound vinculin become accessible for actin filaments (Janssen et al., 2006). Thus, vinculin-dependent recruitment of additional F-actin fibers strengthens the endothelial integrity in response to higher hemodynamic forces.

In ECs, VE-cadherin was shown to conduct shear stress-induced mechanotransduction from AJ to TJ in order to stabilize the endothelial barrier in response to increased mechanical forces (Walsh et al., 2011). T-cell lymphoma invasion and metastasis-inducing protein 1 (TIAM1) mediates signal transmission from AJ to TJ through activation of Rac1 followed by decrease in tyrosine phosphorylation of occludin and increase in TJ assembly (Singleton et al., 2005).

In the endothelium, mechanical stress leads to the activation of multiple signaling protein kinases including Src, v-akt murine thymoma viral oncogene homolog (Akt), extracellular signal-regulated kinase (Erk), Jnk (c-jun N-terminal kinase), and VEGF receptor 2 (VEGFR2; Davies, 1997). The AJ-associated mechanosensory complex comprising VE-cadherin, VEGFR2, and CD31 is primarily responsible for mechanotransduction of shear stress-induced signaling through the cytoskeleton. In EC lines deficient for either CD31 or VEGFR2, no activation of integrins and placement of actin fibers along the flow direction were observed (Tzima et al., 2005). In the mechanosensory complex, VE-cadherin serves as an adaptor that transmits shear stress-induced stimuli to VEGFR2 that in turn activates PI3K (Conway et al., 2013). β-Catenin seems to be involved in mediating assembly between VEGFR2 and VE-cadherin since β-catenin-deficient ECs loss the ability to initiate shear stress-dependent integrin activation (Chervin-Pétinot et al., 2012).

In atheroprone arterial sites, disturbed blood flow (low shear stress or perturbed flow) could induce local proinflammatory activation of ECs through CD31-dependent activation of the mechanosensory complex that leads to induction of nuclear factor-κB (NF-κB), a transcription factor that directs expression of many proinflammatory genes (Harrison et al., 2013). In areas of low shear stress, inflamed endothelium attracted leukocytes increasing their recruitment. In ApoE-deficient mice lacking CD31, increased lesion burden was observed in areas of laminar flow while reduced plaque formation occurred in areas of disturbed flow (Harrison et al., 2013). In a murine model of atherosclerosis, endothelial CD31 was up-regulated in the atheroprone regions with disturbed flow inducing local inflammation and atheroma progression (Goel et al., 2008; Harry et al., 2008). These observations indicate that disturbed hemodynamic forces in atherosusceptible arterial regions could increase risk of preferential plaque formation through stimulation of local vascular inflammation, destabilization of the barrier function, increased leukocyte recruitment and transmigration. Up-regulated endothelial CD31, an important mechanosensor, plays a key role in mediating shear stress-dependent proatherogenic effects.

## Endothelial tubule could be potentially targeted in vessel failure

Vasoactive compounds and mechanical stress (mechanical stretch and shear stress) activate the endothelium. Imbalance between vasoactive substances toward increase in vasoconstrictors and decrease of vasodilators as well as chronic disturbances in hemodynamic forces caused by hypertension, dyslipidemia or diabetes lead to the endothelial dysfunction and increases cardiovascular risk. In ECs, microtubules are one of main cytoskeletal constituents. They are responsible for a variety of functions that are involved in preserving endothelial integrity (Lee and Gotlieb, 2003).

In ECs, microtubules contribute to maintaining cell shape, adhesion, migration, mitosis, and intracellular transport. EC migration is necessary for formation of new vessels and vascular repair. Microtubules are long, hollow cylinders made up of polymerized α- and β-tubulin dimers. Microtubules are dynamic structures due to their ability to assembly and disassembly (Mitchison and Kirschner, 1984). Tubulin is subjected to multiple posttranslational modifications. These modifications are critically contribute to maintaining mucrotubule dynamics and associated functions. In quiescent cells, α-tubulin is acetylated on lysine 40, a hallmark of stable microtubules, In migrating cells, lysine 40 of α-tubulin is deacetylated (Lim et al., 1989). Hyperacetylation of microtubules results in increased microtubular stability and limited cell mobility (Tran et al., 2007). Indeed, the level of tubulin acetylation reflects dynamic changes in microtubule function in response to extracellular signals.

Arterial endothelium is exposed to mechanical stress caused by the bloodflow. Excessive and disturbed mechanical forces could predispose to atherosclerosis. In ECs, mechanical stretch promotes ROS production and contributes to the reorganization of integrins and cytoskeleton (Pandithage et al., 2008). Hemodynamics including shear stress and cyclic stretch is implicated in the modulation of the renin-angiotensin system. Cyclic stretch stimulates production of angiotensin II, a powerful peptide vasoconstrictor, by ECs (Delli Gatti et al., 2008). In the circulation, elevated levels of angiotensin II are associated with related to higher cardiovascular risk (Schmieder, 2007).

Cyclic stretch and angiotensin II were found to be involved in microtubule reorganization since both induce deacetylation of tubulin in ECs. Deacetetylase sirtuin 2 (SIRT2) mediates angiotensin II-dependent microtubule deorganization resulted in increased EC motility (Hashimoto-Komatsu et al., 2011). Furthermore, cyclic stretch could enhance destabilizing influence of angiotensin II on the endothelial integrity. Since angiotensin II type 1 receptor inhibitors prevent angiotensin II-mediated microtubular reorganization, vascular failure initiated by angiotensin II at least in part could arise from the endothelial dysfunction associated with microtubule restructuring (Hashimoto-Komatsu et al., 2011). In ECs, molecular mechanisms of tubulin acetylation/deacetylation are not precisely understood and need further investigations. Histone deacetylase 6 (HDAC6) and SIRT2 are truly responsible for the control of microtubule assembly/disassembly through deacetylation of tubulin (Zhang et al., 2003; Hashimoto-Komatsu et al., 2011). β-adrenergic receptor kinase (BAPK) expressed in ECs and fibroblasts is a recently found activator of HDAC6 that induces increased cell mobility (Hirase and Node, 2012). Elevated levels of epinephrine are known to contribute to hypertension and endothelial dysfunction through the mechanisms involved enhanced vasoconstriction. Indeed, finding a link between BAPK/HDAC6 may help in discovering new pathways by which enhanced sympathetic activity may induce the endothelial dysfunction.

## Concluding remarks

Endothelial dysfunction, a first step of vascular disease, affects other vascular cells such as vascular smooth muscle cells (VSMCs) and immune cells that could finally result in vascular failure. Disruption of endothelial intercellular contacts impairs the proper function of the endothelial barrier, disturbs endothelial integrity, and increases vascular permeability. This promotes vascular failure and supports pathogenesis of vascular disease including atherosclerosis. Posttranslational modifications of junctional proteins including phosphorylation/dephosphorylation could determine stability of intercellular junctions between ECs and are crucially involved in the regulation of vascular permeability. Indeed, kinases and phosphatases specific for phosphorylation and dephosphorylation of junctional proteins could represent critical players in the control of endothelial permeability (Staddon, 2001; McLachlan and Yap, 2007; Hirase and Node, 2012). Chronic cardiometabolic and mechanical stress lead to reorganization and functional changes in the cytoskeleton of ECs. Changes in biochemical properties of EC microtubules are involved in endothelial dysfunction that might be followed by vascular failure. Indeed, more precise investigation of the processes that lead to the endothelial dysfunction could be beneficial in the identification of novel therapeutic targets in cardiovascular-related failure.

### Conflict of interest statement

The authors declare that the research was conducted in the absence of any commercial or financial relationships that could be construed as a potential conflict of interest.
